# Bone wax spacers increase induced membrane thickness and promote osteogenesis more than polymethylmethacrylate spacers in the masquelet technique

**DOI:** 10.1007/s00068-025-02911-w

**Published:** 2025-06-27

**Authors:** Takushi Nakatani, Hajime Mishima, Sho Totsuka, Ryunosuke Watanabe, Norihito Arai, Yohei Tomaru, Hisashi Sugaya, Tomofumi Nishino, Masashi Yamazaki

**Affiliations:** 1 Department of Orthopedic Surgery, Institute of Medicine, University of Tsukuba, Tennodai 1-1-1, Tsukuba, Ibaraki, Japan; 2 Department of Orthopedic Surgery, Showa General Hospital, Hanakoganei 8-1-1, Kodaira, Tokyo, Japan

**Keywords:** Masquelet technique, Induced membrane, Bone wax, Rat, Bone defect, Foreign body reaction

## Abstract

**Purpose:**

The Masquelet technique is a recent novel treatment for severe bone defects. Polymethylmethacrylate (PMMA) has been used as a spacer for bone defects, but the optimal spacer is still unknown. Therefore, this study aimed to histologically evaluate the membranes induced by bone wax and PMMA spacers and compare them with respect to bone formation.

**Methods:**

In this study, bone defects were created in the femurs of Sprague–Dawley rats, and bone wax and PMMA spacers were implanted into the defects to histologically evaluate the induced membrane and bone formation after bone grafting.

**Results:**

As a result, the induced membrane formed by bone wax spacers was significantly thicker than that formed by PMMA spacers, and the vascular area ratio was significantly higher. In addition, bone wax spacers promoted bone formation more than PMMA spacers.

**Conclusion:**

Bone wax spacers promoted induced membrane formation and bone formation by enhancing inflammatory responses more than PMMA spacers.

## Introduction

The treatment of severe bone defects is challenging. Factors contributing to severe bone loss include resection of bone tumors, chronic osteomyelitis, and severe open fractures. Autologous bone grafting is the gold standard for treating bone defects [1]. Autologous bone grafts are ideal for the treatment of bone defects because of their osteoconductive, osteoinductive, and osteogenic properties [2]. Although autogenous bone grafting is a useful treatment modality, large bone defects can lead to resorption of the grafted bone, resulting in poor outcomes.

In 2000, Masquelet reported a novel treatment for severe bone defects [3]. The Masquelet technique is a two-step procedure. In the first step, the bone defect is filled with polymethylmethacrylate (PMMA) bone cement as a spacer. An induced membrane is formed around the PMMA spacer by the foreign body reaction. After 4 − 6 weeks, the PMMA spacer is removed, and an autogenous cancellous bone graft is performed on the defects. Although PMMA spacers are the primary option in the Masquelet technique [4–7], it is not yet known if there are more effective alternatives.

Bone wax is widely used as a hemostatic agent in bone procedures [8]. However, inflammation and fibrous granuloma formation due to foreign body reactions are frequently reported as side effects of bone wax use [9–11]. We hypothesize that the induced membrane formed by bone wax has a different morphology than the membrane formed by PMMA spacers and promotes bone healing after bone grafting.

## Methods

### Animal experiments

This study was approved by the University of Tsukuba Animal Experiment Committee (Approval number: 23–112). All animals were properly treated according to the animal care and control regulations of the University of Tsukuba. Ten-week-old Sprague–Dawley rats (SLC, Shizuoka, Japan) weighing 316–472 g were used in this study. The rearing conditions were 14 h per day (05:00 − 19:00) and 10 h per night (22 ± 1 °C). All rats had free access to food and water, and their body condition was checked weekly. Up to three rats were housed in each cage.

### Spacers

**Fig. 1:**
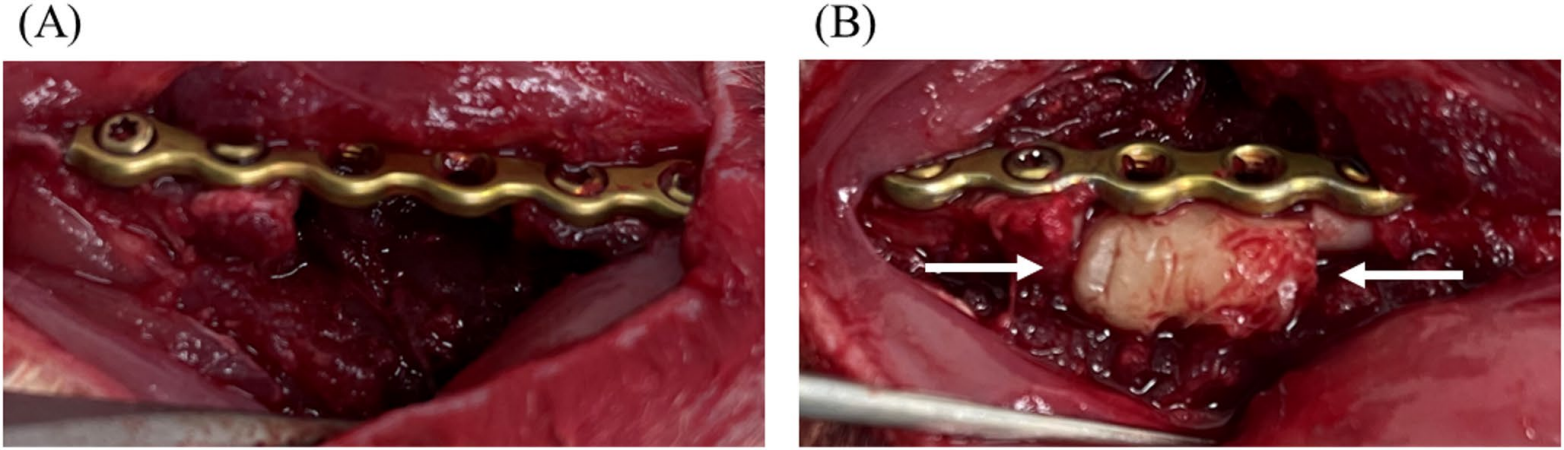
**A** The right femur was plate-fixed, creating a 10-mm defect. **B** Bone wax was inserted into the defect. Care was taken not to push the bone wax into the marrow cavity

All PMMA and bone wax used for the spacers were commercially available; the PMMA was high-viscosity bone cement (PalacosR, Heraeus, Hanau, Germany), and the bone cement was prepared by hand mixing the component monomers and polymers at room temperature (22 ± 1 °C) and molded into a 5-mm diameter cylinder to fit the bone defect. Bone wax (TOKYO M.I. COMPANY, Tokyo, Japan) was hand-formed into a cylindrical shape to fit the bone defect and then implanted. It was not pressed into the medullary canal (Figure 1).

### Surgical procedures

For right femur surgery, rats were placed in the left lateral decubitus position under intraperitoneal general anesthesia (ketamine hydrochloride, 50 mg/kg; xylazine, 1 mg/kg), and the right leg was shaved and cleaned. The skin and fascia were incised longitudinally, and the biceps brachii and vastus lateralis muscles were dissected subperiosteally to expose the femur. A 6-hole, 1.5-mm titanium plate (Synthes, Dübendorf, Switzerland; VA-LCP Hand Plate) was placed on the anterior surface of the femur; two 1.5-mm cortical screws were used to secure the plate proximally and distally. A segmental bone defect was created using a bone saw, and a spacer was inserted. The muscle around the spacer was sutured using a 3 − 0 absorbable suture to prevent dislocation of the spacer. The fascia and skin were sutured using 3 − 0 absorbable sutures, and after subcutaneous injection of cefazoline 30 mg/kg (Otsuka Pharmaceutical, Kanda, Japan), the animals were returned to the cage and observed for any abnormalities. A similar procedure was performed on the left thigh in the right lateral decubitus position.

### Sacrifice

For euthanasia, animals were placed in a CO_2_ chamber and exposed to excessive amounts of CO_2_. Respiratory arrest was confirmed visually, and cardiac arrest was confirmed by palpation.

The plate was removed, and for PMMA spacers, the induced membrane was resected from the femur and cut longitudinally. The spacer was then removed from the membrane. The induced membrane formed by the PMMA spacer was wrapped around bone wax and fixed with 4% paraformaldehyde (PFA). After fixation, the tissue was embedded in paraffin. For bone wax-induced membranes, the membrane was excised en bloc after the implant was removed. To evaluate bone formation, the entire femur was removed and fixed intact in 4% PFA for demineralization. After demineralization, the implant was removed and embedded in a paraffin block.

### Experiment 1: comparison of induced membrane

**Fig. 2:**
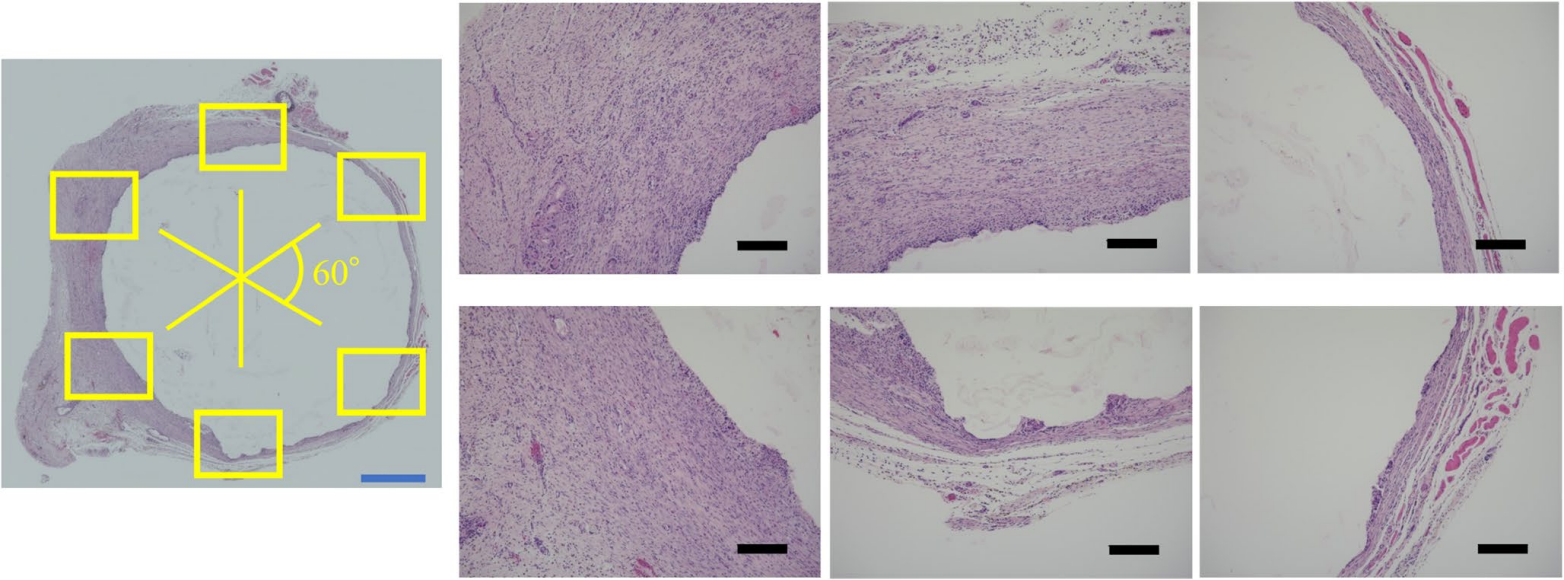
Histological evaluation. The entire circumferential membrane was photographed in a 4× field of view, and the tissue evaluation site was determined; six fields of view were determined, shifted by 60°, and photographed again in a 10× field of view and output to a TIFF file. The output image data was imported into ImageJ for histological evaluation. Blue bar = 1000 μm, Black bar = 200 μm

Twenty-four rats were randomly divided into bone wax and bone cement groups. A 10-mm critical-size bone defect was created in the right femur, and a spacer was inserted. Induced membranes were removed at 3 and 6 weeks postoperatively. Paraffin-embedded membranes were cut into 3 μm sections and stained with hematoxylin and eosin. A computerized imaging system (BZ-X710, KEYENCE, Osaka, Japan) was used to image the observed tissue at 4× magnification. Six images at 10× magnification were taken every 60° for tissue evaluation (Fig. 2). Image analysis was performed using ImageJ [12]. Membrane thickness was assessed at three locations per image separately for the inner and outer layers [13–15]. To assess the number of blood vessels in the induction membrane, vascular endothelial cells were immunostained using an anti-CD31 antibody, and the ratio of vessel area/membrane area was calculated. The primary antibody was anti-CD31 (overnight, 4 °C, ab182981, 1:300 dilution; Abcam, Cambridge, UK), and secondary antibodies were used for immunostaining (Histofine Simple Stain rat MAX-PO, Nichirei Bioscience, Tokyo, Japan).

### Experiment 2: comparison of osteogenesis

A 5-mm bone defect was created in both femurs of seven rats. A bone wax spacer was implanted in the right femur, and a PMMA spacer in the left femur. Allografts of cortico-cancellous bone from the pelvis of the same old rat were performed under general anesthesia after 3 weeks. Plain radiographs of both femurs were taken at 4 and 8 weeks after bone grafting using a DEXICO 3000DX (DEXICOWIN, Kobe, Japan). Radiographs were taken from a direction perpendicular to the inserted plate at a tube voltage of 60 kVp, tube current of 2 mA, and exposure time of 0.1 msec. Radiographs were scored for osteogenesis, cross-linking, and remodeling using the modified Lane and Sandhu score [16]. Animals were sacrificed 8 weeks after bone grafting, and computed tomography (CT) imaging was performed using a Latheta^®^ CT-200 (Hitachi ALOKA, Tokyo, Japan) at a tube voltage of 50 kVp. Bone graft areas were segmented using computer-aided medical imaging software (3D-Slicer, ITK-SNAP, etc.) [17]. The images were converted to 8-bit images using ImageJ, and the bone threshold was set automatically. The converted images were analyzed using the BoneJ plugin to automatically measure bone volume/total volume (BV/TV) and mean trabecular bone thickness [18]. To evaluate bone formation, the entire femur was removed and fixed intact in 4% PFA for demineralization. Paraffin-embedded femurs were sectioned at 3 μm and stained with hematoxylin-eosin, safranin 0, and Masson’s trichrome stain for histologic evaluation of bone formation and endochondral ossification in bone defects using the imaging system described above. Orthopedic surgeons (TN and ST) evaluated all specimens.

### Statistical analysis

All statistical analyses were performed using EZR (Saitama Medical Center, Jichi Medical University, Japan), a graphical user interface for R (The R Foundation for Statistical Computing, Vienna, Austria, version 3.6.1) [19]. The Kruskal–Wallis test, a nonparametric analysis, was used to compare the induced membrane groups by comparing the inner and outer membrane thicknesses and the vessel lumen ratio in each group. The Steel–Dwass method was used for multiple testing between groups, and Wilcoxson’s signed rank test was used to evaluate osteogenesis on plain radiographs and CT, with statistical significance set at *p* < 0.05.

## Results

**Fig. 3:**
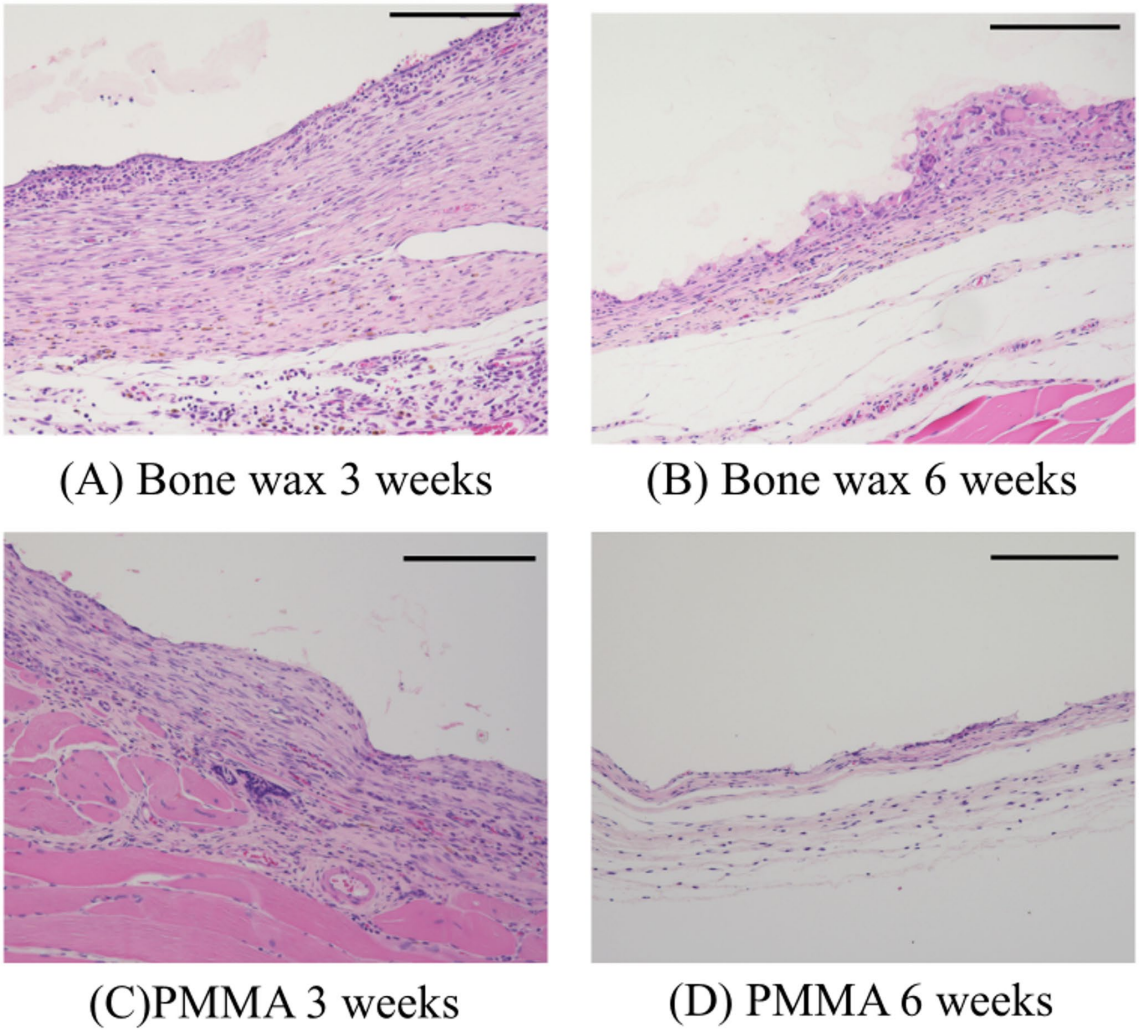
Hematoxylin-eosin staining of the induced membrane. The tissue shows a dense inner layer of cells and a thick outer layer of fibroblasts on the surface in contact with the spacer. The membrane thinned from 3 to 6 weeks, regardless of the type of spacer. Bar = 200 μm

**Fig. 4:**
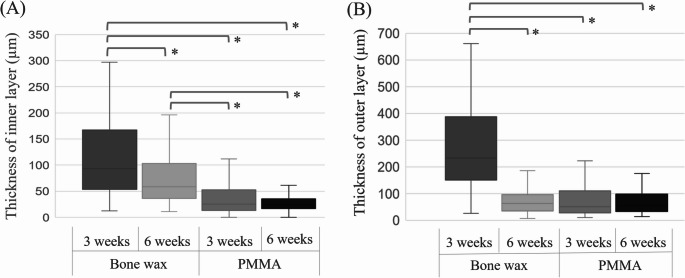
Evaluation of induced membrane. **A** Inner layer thickness: The thickness of the inner layer of the induced membrane was 93 μm ± 153 (standard deviation) and 59 μm ± 57 (SD) at 3 and 6 weeks, respectively, for bone wax and 25 μm ± 61 (SD) and 23 μm ± 44 (SD) at 3 and 6 weeks, respectively, for PMMA. Comparison between groups showed that bone wax at 3 weeks was significantly thicker than the other groups. Bone wax at 6 weeks was significantly thicker than the PMMA group at 3 and 6 weeks. * *p* < 0.01. **B** Outer layer thickness. The thickness of the outer layer of the induced membrane was 232 μm ± 150 (SD) and 64 μm ± 62 (SD) at 3 and 6 weeks, respectively, for bone wax and 50 μm ± 210 (SD) and 54 μm ± 56 (SD) at 3 and 6 weeks, respectively, for PMMA. The 3-week bone wax group had significantly thickened outer layers compared to the other groups. * *p* < 0.01

**Fig. 5:**
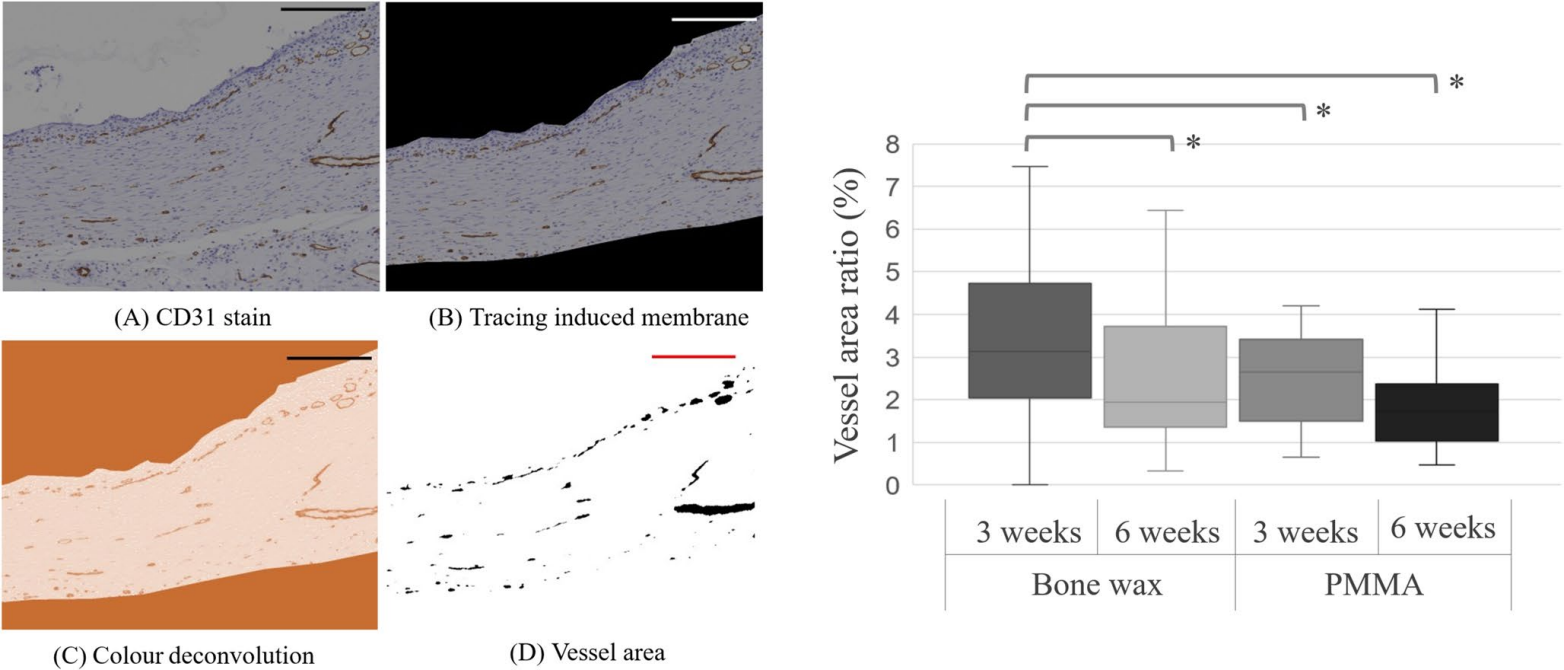
Vascular lumen assessment. **A** Immunostained image of CD31 antibody. **B** Manual extraction of the induced membrane and erasing of the other areas. **C** Using the color deconvolution function of imageJ, vector [H DAB] was selected and images were divided into three colors using color2 (R:0.26814753, G:0.57031375, B:0.77642715). (D) The threshold of the selected image was set to 15–170 to extract CD31-positive cells, which are vascular endothelial cells. If the upper limit of the threshold was set at 170, some non-CD31-positive cells were also contained. Vessel areas were filled. The minimum of [Filter] was set, and only the vessel lumen is plotted. Bar = 200 μm. *p < 0.05

Membrane sampling was possible in all cases. However, one of the PMMA specimens had inadequate tissue fixation at 6 weeks, making tissue evaluation difficult. In all cases, both the PMMA and bone wax groups showed a two-layered structure of the induced membrane (Fig. 3). The median thickness of the inner layer was 93 μm for bone wax and 26 μm for PMMA at 3 weeks postoperatively (*p* < 0.01) and 58.5 μm for bone wax and 23 μm for PMMA at 6 weeks postoperatively (*p* < 0.01), with the bone wax spacer inducing a significantly thicker membrane than the PMMA spacer. The median thickness of the outer layer was 232 μm for bone wax and 50 μm for PMMA at 3 weeks (*p* < 0.01), indicating a significant difference, while at 6 weeks, the median thickness was 63.5 μm for bone wax and 54 μm for PMMA, indicating a non-significant difference, but a trend toward a thicker layer was observed for bone wax (Fig. 4). The lumen-to-vessel ratio of the membrane by CD31 immunostaining was 3.4% for bone wax and 2.8% for PMMA at 3 weeks postoperatively, indicating a significant increase in vascularity in the bone wax (*p* < 0.05; Fig. 5).

**Fig. 6:**
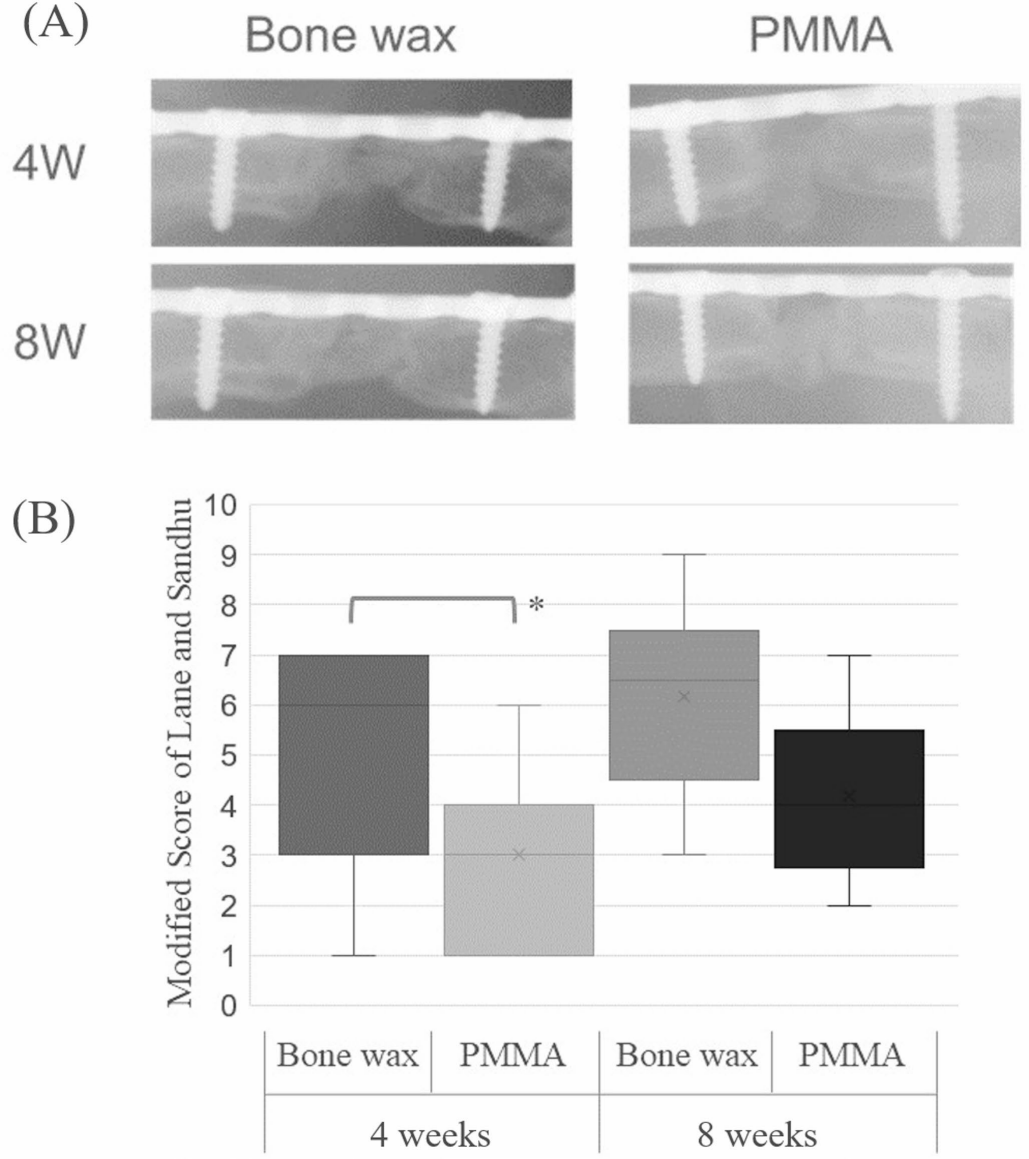
**A** Assessment of osteogenesis by radiographs. **B** The median values at 4 weeks after bone grafting were 6 ± 2.2 (standard deviation [SD]) and 3 ± 1.8 (SD) in the bone wax and PMMA groups, respectively; the values at 8 weeks after bone grafting were 6.5 ± 2.0 (SD) and 4 ± 1.7 (SD) in the bone wax and PMMA groups, respectively. At 4 weeks after bone grafting, bone formation was significantly greater in the bone wax group, *p < 0.05

**Fig. 7:**
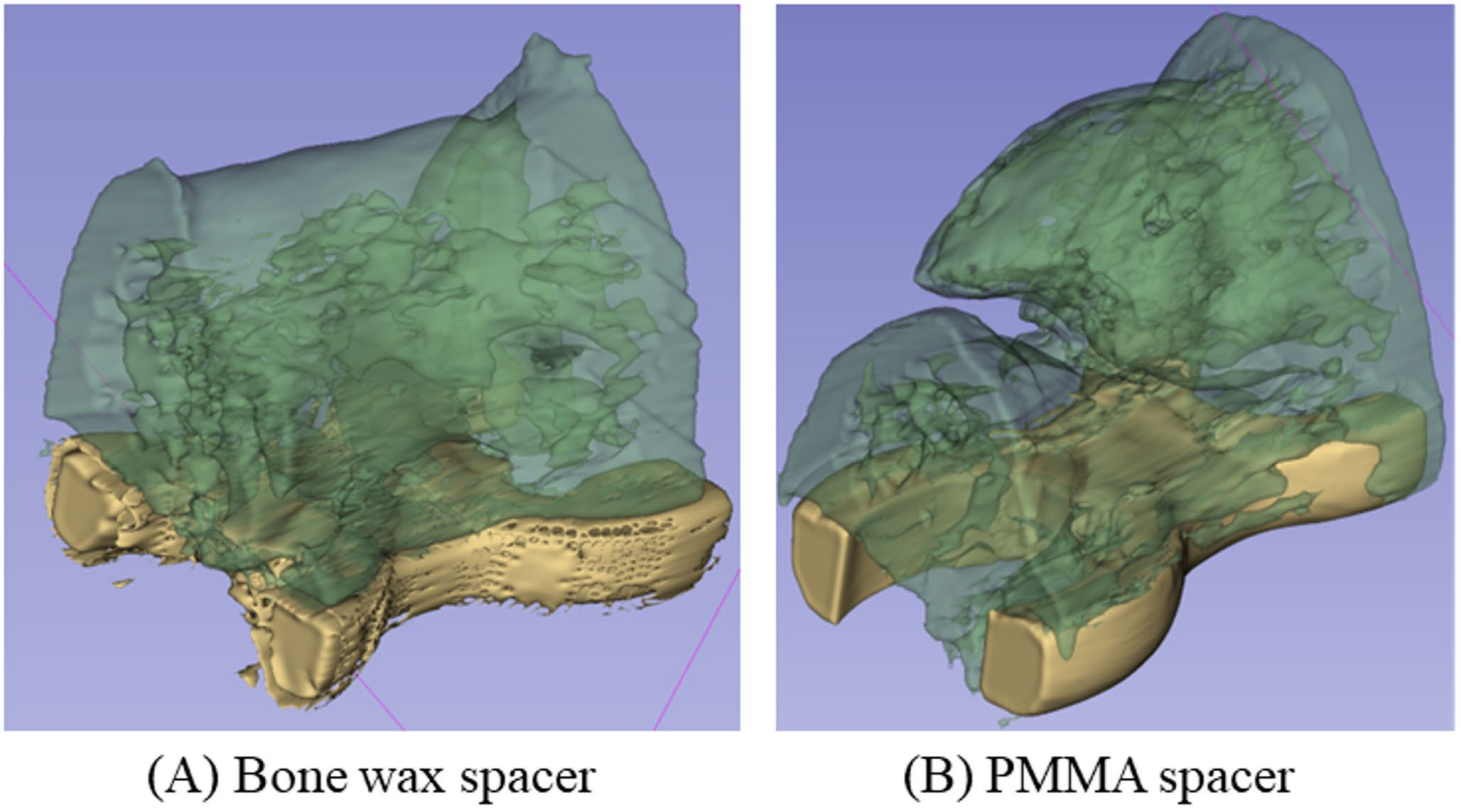
CT evaluation of bone formation in the explanted bone. This 3D image shows the segmentation of the bone defect only. In the bone wax spacer, the allograft bone is fully cross-linked, the edges of the allograft bone become cortical bone, the center is trabecular bone, and the allograft bone remodels to form a medullary cavity. Osteogenesis of the allograft bone is also observed in the PMMA spacer, but not to the point of complete cross-linking formation

**Fig. 8:**
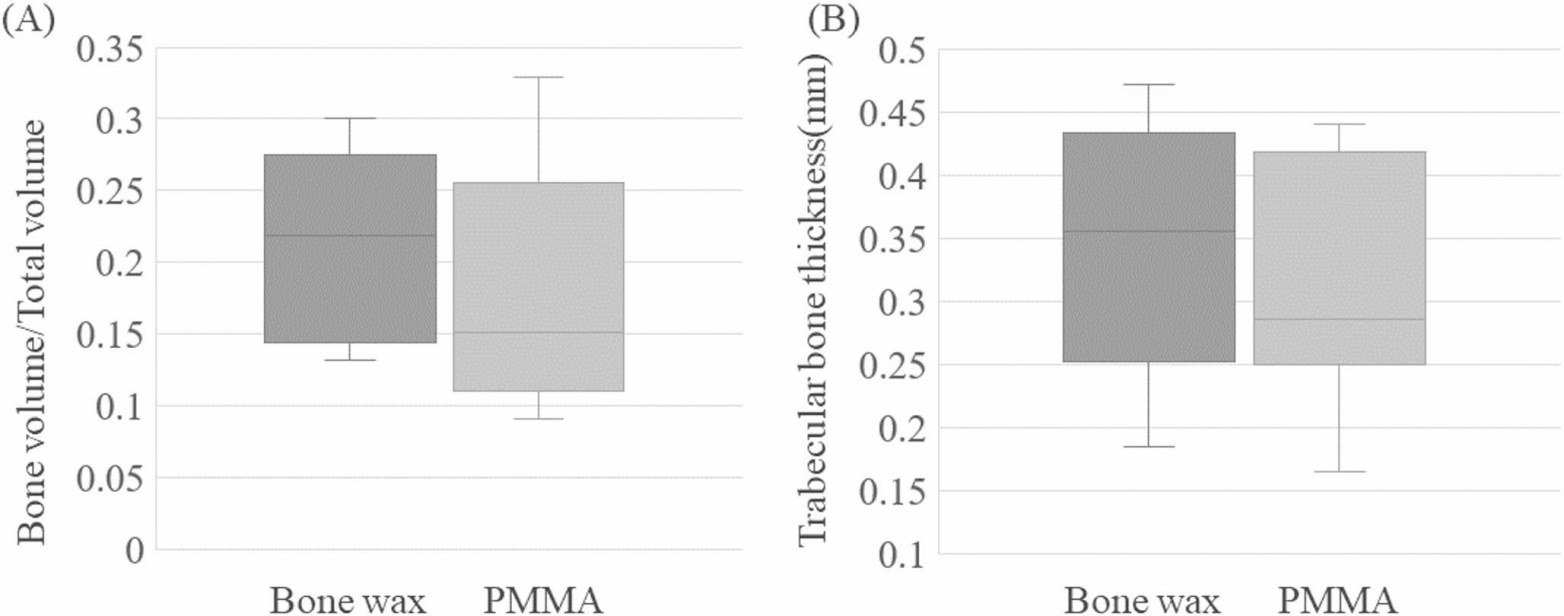
**A** Bone volume/total volume of bone graft was a median of 0.22 ± 0.09 (standard deviation [SD]) and 0.16 ± 0.09 (SD) in the bone wax and PMMA groups, respectively. **B** Trabecular bone thickness of the grafted bone was a median of 0.36 ± 0.10 (SD) and 0.29 ± 0.10 (SD) in the bone wax and PMMA groups, respectively, both showing a trend toward bone formation in the bone wax group compared to the PMMA group but no significant difference

Radiographic evaluations allowed imaging in all cases, but, in one case, infection and implant loosening were observed at 8 weeks after bone grafting, and this case was excluded. Bone formation progressed over time in all rats, with modified Lane and Sandhu scores of 6.0 and 3.0 in the bone wax and PMMA groups, respectively, at 4 weeks (*p* < 0.05), indicating significant bone formation. At 8 weeks, the scores were 4.0 and 6.5 in the PMMA and bone wax groups, respectively, although the difference was not significant, and the score tended to be higher in the bone wax group (Fig. 6). CT evaluation of the harvested bone showed that the BV/TV was 0.22 for bone wax and 0.15 for PMMA, and the mean trabecular bone thickness was 0.36 mm for bone wax and 0.29 mm for PMMA. Although osteogenesis tended to be more advanced in the bone wax group, there was no significant difference between the two groups (Figs. 7 and 8).

**Fig. 9:**
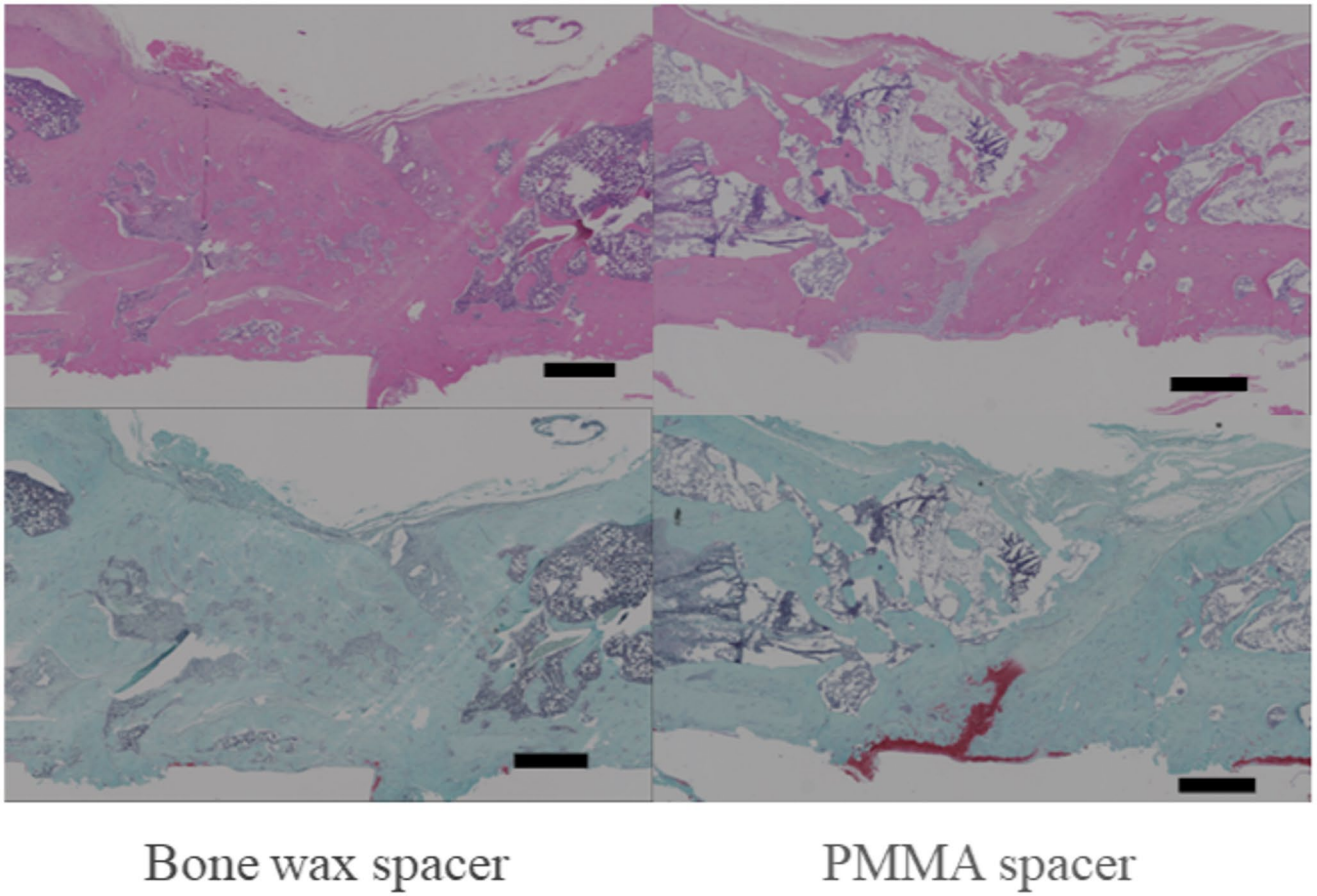
Histological evaluation of the harvested bone. Top row: hematoxylin-eosin stain, bottom row: safranin O stain, bar = 1000 μm

Histological evaluation of the bone grafted area confirmed that the free grafted bone was viable, as osteocytes were observed in the osteoblasts within the grafted bone in both the PMMA and bone wax spacer groups. Therefore, it is believed that the allograft bone grafted within the induced membrane was present without bone resorption due to increased revascularization from the membrane of both spacers. Specifically, in the bone wax group, the allograft bone cross-linked, and the center of the medullary cavity became partially cancellous bone, and remodeling progressed. In addition, new bone was observed extending into the bone defect from both ends of the defect along the guiding membrane. In particular, chondrogenesis was observed on the bone surface and in the bone defects with Safranin O staining. We believe this indicates that the mode of ossification associated with cross-linking in the observed bone defects is endochondral ossification (Fig. 9).

## Discussion

This is the first preliminary study using bone wax as a spacer in the Masquelet technique, which demonstrated that bone wax spacers thickened the induced membrane more than PMMA spacers, promoted angiogenesis, and even enhanced osteogenesis. The induced membrane forms as a result of an inflammatory response due to a foreign body reaction to the PMMA spacer in the Masquelet technique [20, 21]. In the present study, the induced membrane that was formed around the bone wax spacer 3 weeks after implantation was thicker and showed more extensive angiogenesis than the membrane induced by the PMMA spacer. The thickening of the membrane was attributed to inflammatory cell aggregation, tissue swelling, and fibroblast proliferation, and the bone wax spacer appeared to enhance inflammatory response than the PMMA spacer. This is because macrophages, which play an important role in the foreign body response, are approximately 10–20 μm in diameter, and cells perceive larger surface features as flatter [22]. Therefore, the degree of inflammation depended on the roughness of the recognized material surface, suggesting that the composition of the membrane in the acute phase was altered by the spacer. In the initial phase, up to 2 weeks after spacer implantation, the induced membrane histologically shows a two-layered structure consisting of an inner layer of dense cells adjacent to the spacer and an outer layer of fibroblasts and collagen fibers surrounding the inner layer [23, 24]. Induced membranes are rich in mesenchymal stem cells, and the presence of osteoblasts within the membrane has been reported [15]. There are also reports of bone and cartilage formation inside and outside the membrane prior to bone grafting [25]. In other words, the induced membrane not only promotes revascularization of the grafted bone but also provides a mechanism by which mesenchymal stem cells within the membrane can differentiate directly into bone. However, the requirements for this and the mechanisms involved are still unknown [5, 26]. After 3 weeks, a three-layered structure is reported, with thick, denuded vascular tissue forming in the outer layer. Durand et al. reported that the number of nuclei in the induced membrane was low, and the membrane was thin in cases of treatment failure using the Masquelet technique [21]. It is important to have a thick membrane and an abundance of fibroblasts in the first stage of the Masquelet procedure, as well as a strong inflammatory response induced by the bone wax spacers. The presence of fibroblasts and mesenchymal stem cells in the membrane, along with their secretion of growth factors and cytokines to promote angiogenesis, is crucial for bone healing. In this regard, bone wax spacer-induced membranes may be advantageous for bone healing compared to PMMA-derived membranes. On the other hand, histological evaluation at 6 weeks postoperatively showed no significant differences in induction membrane thickness or vessel area ratio between the different spacers. Liu et al. reported that membrane thickness peaked at approximately 1 month after implantation and then gradually thinned [27]. Anderson et al. reported that differences in material properties in the foreign body response peaked at 2 − 4 weeks after implantation [28]. Pelissier et al. also reported a decrease in vascular endothelial growth factor (VEGF) expression over time in a rabbit-induced membrane model [24]. In other words, the inflammatory response subsided, resulting in thinner membranes and reduced VEGF production, which was also thought to result in fewer blood vessels in the membranes.

In clinical practice, PMMA is used as a spacer in the Masquelet technique, but there have been reports of histological evaluation of induced membranes formed by different spacers in animal studies. Mathieu et al. used polypropylene syringes as spacers and reported that membranes formed around the PMMA spacer. Furthermore, they reported that a membrane similar in thickness to that formed around the PMMA spacer was formed, along with bone formation comparable to that observed with PMMA spacers [29]. Moreover, De Mones et al. reported using silicon as a spacer and observed the formation of induced membranes similar to those formed with PMMA, as well as similar amounts of mesenchymal stem cells and BMP-2 in the membrane [30]. Toth et al. compared membranes induced by cylindrical titanium and PMMA spacers and found that the titanium spacer with a rougher surface induced the thickest membrane. However, they noted significantly lower bone healing rates compared to PMMA spacers [31, 32].

Radiographs showed significantly higher osteogenesis scores in the bone wax spacer group at 4 weeks after bone grafting. Both groups had higher scores than the PMMA spacer group in the categories of cross-linking and remodeling, although there was no bone resorption in either group. This means that early osteogenesis was noted in the bone wax group. On the other hand, at 8 weeks after bone grafting, the scores tended to be higher in the bone wax group than in the PMMA spacer group, but the difference was not significant. The same was true for the CT scan of the explanted bone. These results suggest that the induced membrane formed by the bone wax may lead to earlier bone formation and shorter time to bone union compared to that formed by PMMA spacer. Histologically, both groups showed osteocytes in the lacunae of the allograft bone, indicating that the allograft bone was viable by hematoxylin and eosin staining. This was a histological confirmation of the results obtained by radiography and CT. Safranin O staining showed cartilage formation on the bone surface for both the bone wax and PMMA spacers. Previous reports have demonstrated a pattern of osteogenesis by endochondral ossification using the Masquelet technique [25, 33, 34]. This would suggest that the same bone repair mechanism exists in both bone wax and PMMA spacers. The present study found that bone wax spacers promoted bone formation more effectively than PMMA spacers in the Masquelet technique. The validation of the efficacy of bone wax spacers will require several experiments. These include an immune-histological analysis of the dynamics of macrophages and bone formation cytokines in the induced membrane derived from the bone wax spacers. Furthermore, additional studies in large animals are required prior to clinical application. This is because, in small animal experiments, the transplanted bone is considered allogeneic bone transplantation. The original Masquelet technique is based on autologous bone transplantation; therefore, the kind of bone used for transplantation may influence treatment outcomes. Furthermore, while it is possible to mix antibiotics with PMMA spacers to prevent infection, it is unclear whether this can be done with bone wax. Basic research is required to determine the release rate of antibiotics when they are incorporated into bone wax.

Other advantages of bone wax spacers include increased reliability of tissue preparation. Typically, induced membrane tissue is embedded in paraffin blocks after spacer removal and formalin fixation. After thin sectioning, the tissue is deparaffinized with xylene and stained with a staining solution. As bone wax is mainly composed of beeswax, which has similar properties to paraffin, it dissolves with paraffin during xylene treatment. Therefore, when bone wax is used as a spacer, it can be fixed in formalin *en bloc* after membrane removal and observed as a circular tissue specimen without losing the shape of the induced membrane.

### Limitations

This study used bone wax as a spacer for large bone defects in rats. However, the original use of bone wax was for hemostatic purposes in bone marrow and trabecular bone, in which case only a small amount of bone wax was used. If this study is translated directly into clinical practice and bone wax is used for large bone defects in human long bones, a very large amount of bone wax will be used. We are concerned about the possibility of an enhanced inflammatory response in such cases. In addition, PMMA spacers are extremely resistant to compressive stress, and while the spacer alone contributes to mechanical stability against loading of the bone defect, bone wax is extremely soft and does not provide mechanical stability. In this study, the mechanical weakness of bone wax spacers may have been a cause of the large variation in induced membrane formation. Therefore, a more stable fixation method or a solution, such as coating the PMMA surface with bone wax, may be necessary.

## Conclusion

The bone wax spacer enhanced inflammation response at the spacer surface and increased the thickness of the induced membrane and the intramembranous vascular lumen ratio compared to the PMMA spacer. In both femoral bone graft models, the bone wax spacer showed a histologically similar endochondral ossification pattern to the PMMA spacer surface and promoted bone formation better than the PMMA spacer.

## Data Availability

No datasets were generated or analysed during the current study.
